# Preventing Childhood Anxiety Disorders: Is an Applied Game as Effective as a Cognitive Behavioral Therapy-Based Program?

**DOI:** 10.1007/s11121-017-0843-8

**Published:** 2017-09-27

**Authors:** Elke A. Schoneveld, Anna Lichtwarck-Aschoff, Isabela Granic

**Affiliations:** Behavioural Science Institute, Radboud University, Montessorilaan 3, 6525 HR Nijmegen, The Netherlands

**Keywords:** Randomized controlled trial, Non-inferiority, Anxiety, Children, Prevention, Applied game, CBT

## Abstract

A large proportion of children experience subclinical levels of anxiety and cognitive-behavioral therapy (CBT) aimed at preventing anxiety disorders is moderately effective. However, most at-risk children do not seek help or drop out of programs prematurely because of stigma, lack of motivation, and accessibility barriers. Applied games have received increased attention as viable alternatives and have shown promising results, but direct comparisons between applied games and the gold-standard CBT are lacking. Our aim was to investigate whether the applied game *MindLight* is as effective as CBT (i.e., Coping Cat) within an indicated prevention context. We conducted a randomized controlled non-inferiority trial with a sample of 174 children (7- to 12-year olds) with elevated levels of anxiety, comparing *MindLight* to CBT. Anxiety was assessed with self- and parent-reports at pre- and post-program, and at 3- and 6-month follow-ups. Intention-to-treat and completers-only confidence interval approach and latent growth curve modeling showed an overall significant quadratic decrease in child- and parent-reported anxiety symptoms over time and, as predicted, the magnitude of improvement was the same for *MindLight* and CBT. The within-group effect sizes were small to medium at post-test (− 0.32 to − 0.63), and medium to large (− 0.60 to − 1.07) at 3- and 6-month follow-ups. Furthermore, *MindLight* and CBT were rated equally anxiety inducing, difficult, and appealing; CBT was rated as more relevant to daily life than *MindLight*. The current study adds to the growing research on applied games for mental health and shows that these games hold potential as alternative delivery models for evidence-based therapeutic techniques.

**Electronic supplementary material:**

The online version of this article (10.1007/s11121-017-0843-8) contains supplementary material, which is available to authorized users.

## Introduction

Anxiety disorders are the most common mental health disorders in childhood, affecting up to 22% of children (Beesdo et al. 2009). A much larger proportion of youth experience subclinical levels of anxiety with prevalence rates up to 49% (Muris et al. 2000a). These anxiety symptoms commence in childhood and show a chronic and disabling course, especially for individuals showing higher severity and persistence of anxiety symptoms (Asselmann and Beesdo-Baum 2015). Left untreated, anxiety symptoms are associated with a lower general quality of life (Ramsawh and Chavira 2016), worse school performance (Owens et al. 2012), and substance use (Pardee et al. 2014). Effective anxiety prevention programs delivered during childhood, before full-blown anxiety disorders develop, are urgently needed.

### Preventing Anxiety Problems

Many anxiety prevention programs are based on cognitive-behavioral therapy (CBT), the first-line treatment of choice for anxiety disorders (James et al. 2015). In CBT, youth are taught to recognize feelings related to anxiety (i.e., emotions and bodily sensations), to identify and challenge anxious self-talk, to develop coping skills, and to evaluate and reward skill use. In addition, youth are exposed to threatening situations and taught to use relaxation techniques in the face of these threats, a key element of CBT (Kendall and Hedtke 2006). Various recent meta-analyses show that anxiety prevention programs that target youth with some degree of risk (i.e., selective or indicated) result in small (e.g., Stockings et al. 2016) to moderate (Mychailyszyn et al. 2012) effect sizes.

Outside of research contexts, however, the majority of children who could benefit from these prevention efforts do not seek help (Salloum et al. 2016) and those who do often dropout of service prematurely (de Haan et al. 2013). Stigma associated with mental health care is a major barrier to delivering conventional treatments (Salloum et al. 2016). Children do not want to be identified as mentally ill and parents fear being blamed for their children’s problems, further preventing children and parents from seeking the help they need (Mukolo and Heflinger 2011). In addition, some families may not be able to afford mental health services (Salloum et al. 2016) or simply have difficulties reaching services due to difficulties in transportation (Green et al. 2012). Thus, pragmatic reasons often hamper the accessibility of conventional prevention programs. Additionally, high dropout rates are a major threat to the effectiveness of conventional (CBT) programs (de Haan et al. 2013), possibly because the programs are not appealing and engaging to children (World Health Organization 2012). These barriers call for a reconsideration of our current group-based and clinical expert-led delivery models of prevention programs (e.g., Kazdin 2015).

### Applied Games for Mental Health

Recently, applied games have received increasing attention as a viable and cost-effective alternative delivery model for prevention efforts (Kazdin 2015). The promise of applied games lies in the intrinsically motivating features of games, their high accessibility, reach, scalability, affordability, and convenience (e.g., Granic et al. 2014). Despite these potential advantages of applied games, reliable outcome evidence from rigorous research designs is needed before these games can be considered evidence-based alternative interventions. Very few studies have tested the effects of applied games according to rigorous scientific standards.

Studies investigating applied games for anxiety that have used randomized controlled trials (RCTs) have shown promising results. *Dojo*, an emotion management video game that helps youth recognize and control their physiological and emotional arousal, has been found to significantly decrease anxiety symptoms in youth with elevated levels of anxiety (i.e., indicated prevention; Scholten et al. 2016). *MindLight* is another applied game specifically designed for children with elevated levels of anxiety. The game uses several evidence-based techniques including neurofeedback (Price and Budzynski 2009), exposure training (Kendall et al. 2005), and attention bias modification (Bar-Haim et al. 2011) which are embedded in a horror-themed survival game that trains children to cope with their anxiety. An initial indicated prevention RCT showed significant improvements in anxiety symptoms after game play and at 3-month follow-up (Schoneveld et al. 2016). However, both the *Dojo* and the *MindLight* trials employed alternative, commercial games as their control condition. The more rigorous test for the effectiveness of these applied games is to demonstrate non-inferiority (i.e., equal efficacy) to the effective gold standard in anxiety prevention: CBT. To date, there are no direct comparisons of applied games for children with elevated levels of anxiety and CBT (Fleming et al. 2017); the current study was designed to fill this gap.

### Current Study

We ran a two-armed randomized controlled non-inferiority trial (Piaggio et al. 2012) comparing *MindLight* to CBT within an indicated prevention context. The aim of the current study was to determine whether *MindLight* was as effective as CBT for children with elevated anxiety symptoms. We choose *MindLight* over *Dojo*, because anxiety symptoms are most prevalent in childhood and *MindLight* is, in contrast to *Dojo*, designed for children. Based on previous indicated prevention RCTs with *MindLight* (Schoneveld et al. 2016) and CBT (van Starrenburg et al. 2017), our primary hypothesis was that children with elevated anxiety symptoms in the *MindLight* condition would show comparable decreases in anxiety symptoms as children in the CBT condition. Further, we aimed to test the effectiveness of the design of the game beyond its impact on anxiety symptoms. Specifically, based on evidence-based exposure principles (Kendall et al. 2005), we tested whether the game elicited the feelings of anxiety that it was designed to trigger, in order for exposure techniques to be relevant. We also examined the game’s motivating properties and appeal to children. Our secondary hypothesis was that children would rate *MindLight* as more appealing compared to CBT but equally anxiety inducing.

## Methods

### Study Design

In eight primary schools in the southeast part of the Netherlands, children were randomized in a multicenter, stratified, parallel group, equivalence study comparing the effect of *MindLight* versus CBT between February 2015 and January 2016. An independent researcher from our research institute carried out the randomization with an allocation ratio of 1:1 within school and stratified by sex and grade. Four separate groups of children were created: younger boys (grades 3 and 4), older boys (grades 5 and 6), younger girls (grades 3 and 4), and older girls (grades 5 and 6). Children within these groups were randomly assigned to *MindLight* or CBT using the SPSS random number generator. The study was approved by the ethics committee of the Faculty of Social Sciences of the Radboud University (EC2013-0410-139a1) and registered at the Dutch Trial Register (www.trialregister.nl; Trial ID: NTR4993).

### Procedure

Participants were recruited in two steps: screening and inclusion. First, all children in grades 3 to 6 from eight primary schools received an information letter for their parents and a screening consent form. All children with active parental permission (*N* = 791) were screened on anxiety symptoms with the child version of the Spence Children’s Anxiety Scale (SCAS; Spence 1998). Second, eligible children were identified by their elevated anxiety symptoms, operationalized based on Muris et al. (2000b): children were eligible if either (a) the total SCAS score was 1 *SD* above the mean or (b) at least two SCAS subscales were 1 *SD* above the mean. This is in line with recommendations by Spence (2013), who defined elevated anxiety symptoms as 1 *SD* above the mean. The obsessive-compulsive disorder subscale was omitted because it is no longer considered an anxiety disorder in the DSM-V. Parents of the 221 (27.9%) eligible children were contacted by phone to inform them about study goals, procedure and programs, to assess exclusion criteria, and to invite them and their child(ren) to participate. Exclusion criteria were currently in anxiety treatment, diagnosis of obsessive-compulsive disorder, post-traumatic stress disorder, or autism spectrum disorder. Initial verbal consent of 174 children was provided. Written informed consent was obtained at pre-test (see below).

The 174 children and their parents were randomly assigned to *MindLight* or CBT. A week prior to the intervention, before they knew to what program they were assigned, children and parents filled out the questionnaires (i.e., pre-test). Parents got a link through e-mail and completed the questionnaire online. Two weeks after intervention termination, children and parents filled out post-test questionnaires. Follow-ups (FUs) were 3 and 6 months after post-test and followed the same procedure as pre-test assessments.

### Sample Size

The target sample size was estimated using the Jones et al. (1996) calculations for equivalence trials. The equivalence margin for improvement in anxiety score was set at 0.16 SCAS points. This difference corresponds to 0.5 *SD* of the anxiety change score (*M* = 0.14, *SD* = 0.32) at post-test in children allocated to CBT, as found in a previous indicated prevention RCT (van Starrenburg et al. 2017). Based on 80% power (1 − β) to detect a clinically relevant difference in improvement of 0.16 points on the SCAS (*α* = .05, two-sided), 50 children were required in each group. To account for attrition, 10% was added and another 25% was added to account for the design effect (based on six children per group and an intraclass correlation of 0.05). In total, this led to a required total sample size of 135 children.

### Participants

**Fig. 1:**
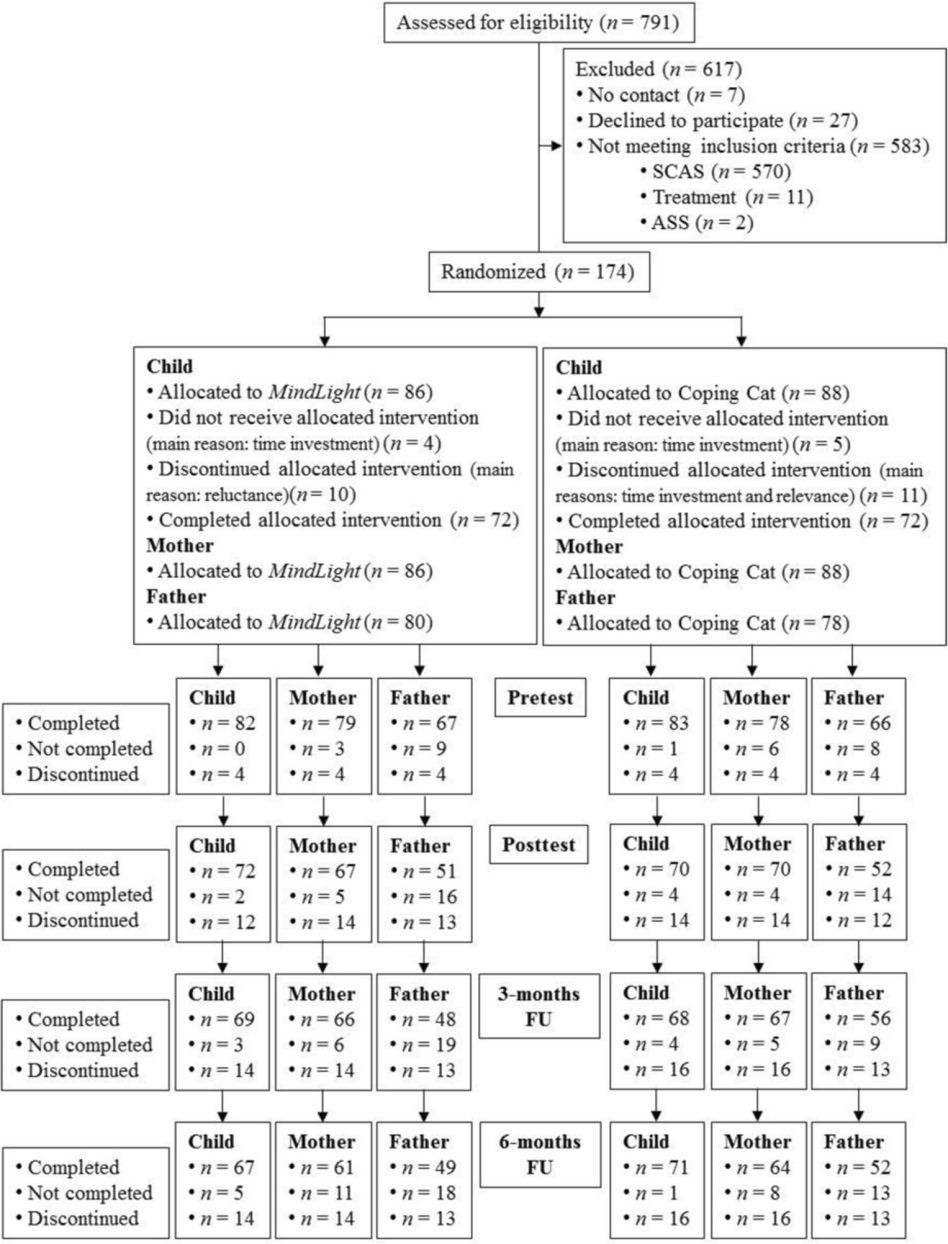
Flowchart of participants through trial

A total of 174 children were randomized (see Fig. 1 for flowchart). At pre-test, children were between 7 and 12 years old (*M* = 9.97, *SD =* 1.16) and 40.8% were boys. The majority of the children were born in the Netherlands (91.4%). Most children attended at least five *MindLight* sessions (*n* = 64; 87.7% excluding dropouts) or at least seven CBT sessions (*n* = 66; 91.7% excluding dropouts). In most cases, both parents participated in the study (*n* = 145). The parent sample included 174 mothers and 145 fathers. At pre-test, mothers ranged in age from 28 to 49 years (*M* = 41.13, *SD* = 3.67), fathers from 33 to 57 years (*M* = 43.49, *SD* = 4.24). The majority of parents were of Dutch descent (87.9% of mothers, 73.6% of fathers).

### Intervention Programs

#### *MindLight*

*MindLight* is a 3D third-person neurofeedback video game produced by the PlayNice Institute (http://theplayniceinstitute.com/) and designed by GainPlay Studio (http://www.gainplaystudio.com/). The game starts with Arty left at the doorstep of his grandmother’s scary mansion faced with the task of saving his grandmother from the evil forces that have possessed her and the house. At his bedroom, he finds his magical glowing hat Teru that teaches him (and the player) to overcome his fears by changing his state of mind. Several theoretically grounded, evidence-based strategies for decreasing anxiety are embedded in the game (i.e., neurofeedback training, exposure training, and attention bias modification), described in detail in Schoneveld et al. (2016). Children control Arty and Teru using a Microsoft Xbox 360 controller and a Neurosky one-channel dry-sensor EEG headset.

Children played *MindLight* for six 1-h sessions, at school after regular school hours every week, except for holidays. Groups consisted of five to ten children and were supervised by Masters students. Children used earplugs to hear the game sound and to diminish distraction. They were seated at least one table away from each other. Supervisors gave instructions about *MindLight* at the beginning of the first session. At the end of the last session, children received a diploma to commemorate their participation in *MindLight*.

#### CBT

Coping Cat is one of the few effective CBT programs for anxious children (Flannery-Schroeder et al. 2005) and was used for the current study. The program teaches children both cognitive (i.e., cognitive restructuring) and behavioral techniques (i.e., relaxation training and exposure). In the current study, a shortened eight-session version of the indicated prevention group-based version of van Starrenburg et al. (2017) was used. We shortened the Van Starrenburg et al. version of Coping Cat according to the content of the American shortened version of the same program (Beidas et al. 2013) in which the problem-solving part was reduced. The first two sessions lasted 1.5 h and the last six sessions lasted 1 h, and took place at schools after regular school hours every week, except for holidays. Groups consisted of four to seven children and were led by two psychologists. Parents received information about the progress of their child and general information about the program halfway through the program and at the end via e-mail. At the end of the last session, children received a diploma to commemorate their participation in CBT.

Psychologists (*n* = 15) had knowledge of and experience with CBT. To prepare, all psychologists successfully completed a 2.5-day training by a certified clinician, in which they received information on the protocol, and practiced exposure techniques and role-playing. Over the course of delivering the program, psychologists participated in 1-h supervision and feedback sessions twice.

### Measures

#### Anxiety Symptoms

Children’s anxiety symptoms were assessed with the child (45 items) and parent (38 items) versions of the SCAS (Spence 1997, 1998). The child version of the SCAS includes seven positive filler items to reduce negative response bias. All items are rated on a 4-point scale: 0 = *never*, 1 = *sometimes*, 2 = *often*, and 3 = *always*. Both the child version (Muris et al. 2000b) and the parent version show good convergent validity (Brown-Jacobsen et al. 2011) and good reliability (Whiteside and Brown 2008). Cronbach’s alpha of the child version was 0.91 at pre-test, 0.90 at post-test, 0.93 at 3 months FU, and 0.91 at 6 months FU. For the parent version, the Cronbach’s alphas were respectively 0.84, 0.80, 0.81, and 0.82 for mothers and 0.83, 0.85, 0.83, and 0.84 for fathers. Four outcome measures were computed: total anxiety, which is the overall mean for child-, mother-, and father-report (except the filler items for the child version) and personalized anxiety, which is the mean subscale score of the subscale that the child scored highest on at screening.

#### Time Spent Playing Games

Children were asked how many hours they play video games on each day of the week. Time spent playing games was calculated by adding these numbers, representing the total number of hours spent playing video games per week.

#### Program Expectations

Expectations about the effect of the program were assessed at pre-test, before the children knew to which condition they were assigned. Children read a short description of both *MindLight* and CBT and answered the following question: to what extent do you think that *MindLight*/CBT will help you to feel less afraid? Children could respond on a scale from 0 to 9, with 0 being “not less afraid,” 5 being “little bit less afraid,” and 9 being “lot less afraid.”

#### Children’s Program Ratings

Children were asked to evaluate the program they were assigned to at post-test and FUs. Children rated the following five statements on a 5-point scale: 0 = *totally disagree*, 1 = *disagree*, 2 = *neutral*, 3 = *agree*, and 4 = *totally agree*. “I found it fun to participate in *MindLight* /CBT”; “I think *__* is fun for other children”; “I can use what I learned from *__* in my daily life well”; “I found some exercises in __ stressful”; “I found some exercises in *__* difficult”. Answers on these questions were analyzed separately.

### Strategy of Analyses

First, to assess baseline differences between the two conditions, we performed *χ* ^2^ tests and *t* tests. Next, *t* tests for independent groups were conducted to examine differences between conditions across time. Tests were performed in IBM SPSS Statistics 21. Second, to test non-inferiority, a two-sided confidence interval (CI) approach was used in both the ITT and CO samples (available online in Table B). Non-inferiority of *MindLight* to CBT could be claimed if the upper bound of the CI for the difference in mean change of anxiety symptoms was below the margin of non-inferiority (Δ = 0.16). Third, latent growth curve modeling (LGCM) was performed in Mplus 7.2 to examine the effect of condition on individual levels of anxiety symptoms at pre-test (i.e., intercept) and changes in anxiety symptoms over time (i.e., slope) in the intention-to-treat (ITT) sample. Missing data were dealt with by multiple imputation (MI), using the Markov chain Monte Carlo method. First, we estimated the initial model based on the four time points (i.e., pre-test, post-test, 3-month FU, and 6-month FU) without any predictors or control variables. Second, we tested whether condition predicted the pre-test levels of anxiety (i.e., intercept) and/or rate of change in anxiety symptoms (i.e. slope). Third, we added participant characteristics (i.e., sex, age, weekly game time, and expectations) to the model and tested whether the interaction between condition and participant characteristics predicted the intercept and/or slope. Results from the LGCM in the completers only (CO) sample are available online in Tables C and D. Lastly, to assess differences between the two programs in children’s ratings, we performed *t* tests for independent groups in IBM SPSS Statistics 21.

## Results

### Descriptive Statistics

Randomization efforts were successful: no differences were found between the *MindLight* and the CBT group on age, weekly game time, expectations and sex (see Table A available online). Therefore, we did not control for these variables in subsequent analyses. In addition, no differences were found between the programs on dropout rates: *χ* ^2^(1) = 0.11, *p* = .740. Means, *SD*s, and *t* values for all anxiety measures at all time points separately for condition are shown in Table 1. Groups did not differ significantly on anxiety symptoms at pre-test, nor any other time point.

**Table 1:** Means, standard deviations, *t* values, and within-group effect sizes of anxiety symptoms and evaluations at all time points separately for programs

Measure	*MindLight*			CBT				*MindLight*			CBT			
	*M*	*SD*	*d*_*av*_	*M*	*SD*	*d*_*av*_	*t* (*df*)	*M*	*SD*	*d*_*av*_	*M*	*SD*	*d*_*av*_	*t* (*df*)
	Pre-test							Post-test						
Anxiety symptoms														
Total child	0.98	0.41		0.99	0.42		0.24 (163)	0.74	0.39	− 0.60	0.75	0.34	− 0.63	0.13 (140)
Personalized child	1.38	0.57		1.31	0.54		− 0.90 (163)	1.07	0.59	− 0.53	1.13	0.48	− 0.35	0.68 (140)
Total mother	0.51	0.26		0.50	0.19		− 0.26 (155)	0.42	0.20	− 0.39	0.42	0.17	− 0.44	− 0.25 (135)
Total father	0.47	0.23		0.46	0.20		− 0.29 (131)	0.40	0.21	− 0.32	0.38	0.18	− 0.42	− 0.53 (101)
Evaluations														
Personal appeal	–	–		–	–		–	2.35	1.39		2.77	1.18		1.94 (139)
Appeal to others	–	–		–	–		–	2.61	1.15		2.59	1.09		− 0.09 (139)
Relevance	–	–		–	–		–	2.13	1.38		2.96	0.95		4.15 (139)^***^
Anxiety-inducing	–	–		–	–		–	2.71	1.39		2.46	1.34		− 1.09 (138)
Difficult	–	–		–	–		–	1.85	1.22		1.99	1.28		0.66 (139)
	3-month FU							6-month FU						
Anxiety														
Total child	0.67	0.42	− 0.75	0.65	0.39	− 0.84	− 0.33 (136)	0.58	0.34	− 1.07	0.64	0.38	− 0.88	1.05 (136)
Personalized child	0.99	0.56	− 0.69	0.93	0.47	− 0.75	− 0.67 (135)	0.86	0.53	− 0.95	0.95	0.50	− 0.69	1.01 (136)
Total mother	0.40	0.21	− 0.47	0.37	0.16	− 0.74	− 1.09 (131)	0.37	0.21	− 0.60	0.34	0.15	− 0.94	− 1.11 (123)
Total father	0.39	0.21	− 0.36	0.35	0.16	− 0.61	− 1.08 (102)	0.34	0.19	− 0.62	0.31	0.17	− 0.81	− 1.00 (99)
Evaluations														
Personal appeal	2.41	1.29		2.62	1.10		1.07 (136)	2.48	1.31		2.55	1.01		0.36 (136)
Appeal to others	2.71	1.04		2.72	1.01		0.08 (136)	2.70	1.10		2.68	0.92		− 0.14 (136)
Relevance	1.96	1.24		2.86	1.00		4.67 (136)***	2.18	1.29		2.58	1.08		1.97 (136)
Anxiety-inducing	2.64	1.24		2.41	1.22		− 1.11 (136)	2.55	1.30		2.32	1.17		− 1.05 (135)
Difficult	1.97	1.29		1.70	1.15		− 1.32 (136)	2.06	1.27		2.11	1.14		0.26 (136)

*Total child* total anxiety child report, *Personalized child* personalized anxiety child report, *Total mother* total anxiety mother report, *Total father* total anxiety father report

*** p < .001

### Non-inferiority

**Fig. 2:**
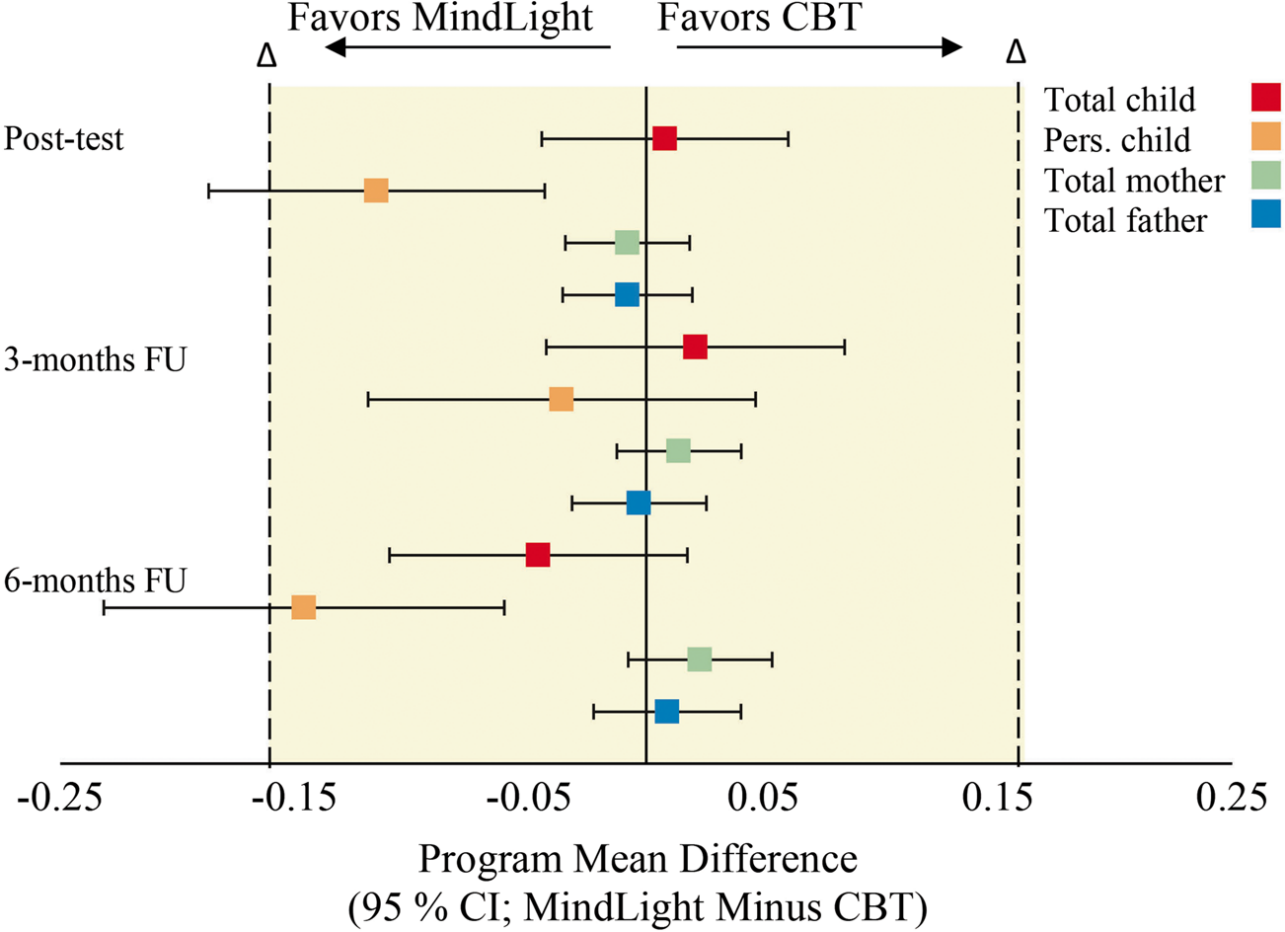
Differences between programs in anxiety symptoms, in relation to non-inferiority

Table 2 presents the change in anxiety symptoms and 95% CIs for both programs over the course of the study. It shows that non-inferiority of *MindLight* to CBT could be demonstrated at post-test, 3-month FU, and 6-months FU for total anxiety child report, total anxiety mother report and total anxiety father report. For personalized anxiety child report, non-inferiority could only be shown at 3-months FU. At post-test and 6-month FU, the CI lay entirely to the left of 0, indicating significant differences in favor of *MindLight*. The results of Table 2 are visualized in Fig. 2.

**Table 2:** Pre-test and change in anxiety symptoms over the study (intention-to-treat sample)

Assessment	*MindLight*	CBT	Mean difference^a^	*SD*	95% CI
Total child					
Pre-test	0.98	0.99			
Post-test–pre-test^b^	− 0.24	− 0.24	0.01	0.34	[− 0.04, 0.06]^c^
3-month FU–pre-test^b^	− 0.32	− 0.34	0.02	0.42	[− 0.04, 0.08]^c^
6-month FU–pre-test^b^	− 0.40	− 0.36	− 0.05	0.42	[− 0.11, 0.02]^c^
*n*	82	83			
Personalized child					
Pre-test	0.98	0.99			
Post-test–pre-test^b^	− 0.31	− 0.20	− 0.12	0.47	[− 0.19, − 0.04]^d^
3-month FU–pre-test^b^	− 0.41	− 0.38	− 0.04	0.54	[− 0.12, 0.05]^c^
6-month FU–pre-test^b^	− 0.52	− 0.37	− 0.15	0.56	[− 0.23, − 0.06]^d^
*n*	82	83			
Total mother					
Pre-test	0.51	0.50			
Post-test–pre-test^b^	− 0.09	− 0.09	− 0.01	0.17	[− 0.03, 0.02]^c^
3-month FU–pre-test^b^	− 0.12	− 0.13	0.01	0.17	[− 0.01, 0.04]^c^
6-month FU–pre-test^b^	− 0.14	− 0.16	0.02	0.20	[− 0.01, 0.05]^c^
*n*	80	81			
Total father					
Pre-test	0.47	0.46			
Post-test–pre-test^b^	− 0.09	− 0.08	− 0.01	0.17	[− 0.04, 0.02]^c^
3-month FU–pre-test^b^	− 0.12	− 0.12	0.00	0.17	[− 0.03, 0.03]^c^
6-month FU–pre-test^b^	− 0.15	− 0.16	0.01	0.19	[− 0.02, 0.04]^c^
*n*	69	69			

*CI* confidence interval

^a^A negative difference is a difference in favor of *MindLight*

^b^A negative score means a decrease in the severity of symptoms

^c^The 95% CI of the difference in symptom change lies entirely between the equivalence margins of − 0.16 and + 0.16 points, indicating equivalence of *MindLight* and CBT

^d^The 95% CI of the difference in symptom change lies entirely to the left of 0, indicating significant differences in favor of *MindLight*

### Latent Growth Curve Modeling

We first fitted a linear growth model with intercept and slope as latent variables for all four anxiety measures separately and found that most model fit indices were unsatisfactory. Second, we added a quadratic term to the growth function. The resulting quadratic growth model with an intercept, a linear slope, and a quadratic slope as latent variables showed a close fit to the data (Table 3). In some cases, the RMSEA value was too high, yet cutoff points of 0.05 and 0.10 are too restrictive for our sample size (Chen et al. 2008) and acceptable models might be unnecessarily rejected. Both the linear and the quadratic slope component were significant for all anxiety measures, indicating that anxiety symptoms decreased significantly over time and that the rate of the decrease slowed over time.

**Table 3:** Initial level (intercept), change (linear slope component) and rate of change (quadratic slope component) in anxiety symptoms on program and moderators (intention-to-treat sample)

	Intercept		Linear slope		Quadratic slope				
	*B*	*p*	*B*	*p*	*B*	*p*	*χ* ^2^ (*df*)	CFI	RMSEA
Quadratic growth model									
Total child	0.98	< .001	− 1.21	< .001	0.97	< .001	11.65 (4)	0.96	0.11
Personalized child	1.35	< .001	− 1.38	< .001	1.07	< .001	8.60 (4)	0.97	0.08
Total mother	0.50	< .001	− 0.45	< .001	0.36	.001	16.65 (4)	0.97	0.14
Total father	0.47	< .001	− 0.41	< .001	0.28	.002	14.59 (4)	0.98	0.13
Program as predictor									
Total child	− 0.02	.760	0.20	.505	− 0.39	.245	3.78 (2)	0.99	0.06
Personalized child	0.07	.337	− 0.09	.821	− 0.17	.737	6.82 (2)	0.98	0.11
Total mother	0.00	.899	0.02	.908	0.04	.826	2.05 (2)	1.00	0.02
Total father	0.01	.823	− 0.04	.778	0.08	.645	2.30 (2)	1.00	0.03
Age as moderator									
Total child	0.04	.423	− 0.13	.480	0.14	.545	3.73 (4)	1.00	0.02
Personalized child	0.01	.884	− 0.00	.993	0.08	.824	6.15 (4)	0.99	0.05
Total mother	− 0.02	.534	0.16	.147	− 0.24	.123	7.01 (4)	0.99	0.06
Total father	− 0.03	.392	0.06	.611	− 0.04	.777	5.90 (4)	1.00	0.05
Sex as moderator									
Total child	0.10	.521	− 0.10	.875	− 0.23	.756	7.42 (4)	0.99	0.07
Personalized child	0.15	.409	− 0.31	.736	− 0.09	.935	14.80 (4)	0.96	0.13
Total mother	0.10	.188	− 0.06	.819	0.17	.586	3.17 (4)	1.00	0.01
Total father	0.14	.050	− 0.17	.564	0.16	.661	2.40 (4)	1.00	0.01
Expectation as moderator									
Total child	− 0.01	.834	− 0.13	.375	0.17	.311	9.34 (4)	0.98	0.09
Personalized child	0.05	.224	− 0.36	.093	0.42	.159	10.66 (4)	0.98	0.09
Total mother	0.01	.514	− 0.02	.758	0.02	.859	7.42 (4)	0.99	0.07
Total father	− 0.02	.206	− 0.03	.727	0.07	.447	8.21 (4)	0.99	0.07
Weekly game time as moderator									
Total child	0.01	.164	− 0.04	.192	0.06	.159	5.99 (4)	0.99	0.05
Personalized child	0.02	.073	− 0.06	.114	0.09	.043	4.73 (4)	1.00	0.03
Total mother	0.00	.859	0.00	.878	− 0.01	.626	4.37 (4)	1.00	0.02
Total father	0.00	.524	0.01	.380	− 0.02	.341	3.90 (4)	1.00	0.02

*Total child* total anxiety child report, *Personalized child* personalized anxiety child report, *Total mother* total anxiety mother report, *Total father* total anxiety father report

**Fig. 3:**
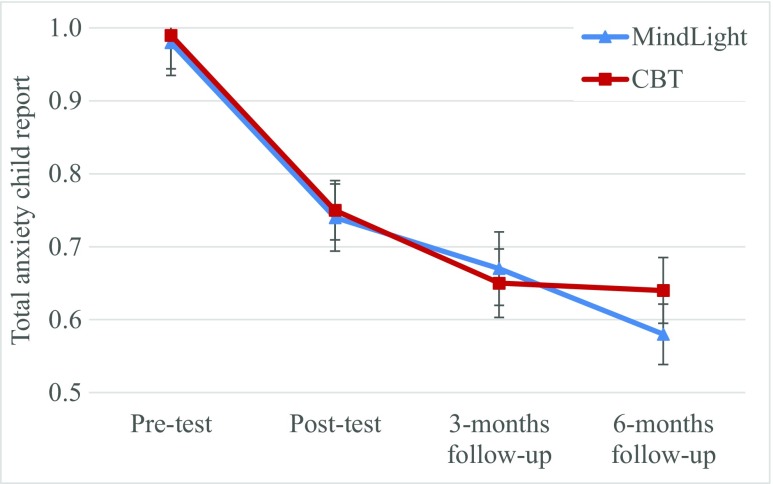
Total anxiety symptoms child report across time by program. Error bars are standard errors

Third, condition was included in the quadratic growth function. Table 3 shows that condition was not related to the intercept, nor the linear, nor the quadratic slope component for all anxiety measures. As predicted, these results indicate that the initial level of anxiety symptoms, the amount of decrease in anxiety measures, and the rate of improvements in anxiety did not differ between conditions. Figure 3 shows the decrease in total child-reported anxiety separate by condition. The pattern in the other models was similar to the one presented in Fig. 3. The within-group effect size for change for all four anxiety measures from pre-test (*d*_*av*_) are small to medium at post-test, and medium to large at 3- and 6-month FUs (Table 1).

Fourth, the interaction between condition and sex, age, weekly game time, and expectations were added separately to the quadratic growth function. Table 3 shows that the interaction between condition and sex predicted the initial level of father reported anxiety symptoms: girls who played *MindLight* showed the highest initial father reported levels of anxiety. Furthermore, the interaction between condition and weekly game time predicted the quadratic slope component of personalized anxiety. This indicates that the rate of decrease in personalized anxiety slowed the most for children who were in the *MindLight* condition and had the highest amount of weekly game time. All other interactions were non-significant.

### Children’s Program Ratings

To compare the children’s ratings of the programs, we conducted *t* tests on the five rating questions (see Table 1). Children who played *MindLight* and children who received CBT rated their program equally appealing to themselves across time points. In addition, at every time point, children in both conditions thought their program was appealing for other children. No differences between the programs were found on reported difficulty nor on the extent to which the programs induced anxiety. Children who received CBT rated the program significantly more relevant to their daily life than children who played *MindLight*.

## Discussion

The current study represents one of the first of a handful of RCTs on applied games for children’s mental health. To date, there have been no other direct comparisons between applied games for anxious children and the CBT gold standard intervention. We aimed to fill this gap by conducting a non-inferiority randomized controlled trial testing equal efficacy of the applied game *MindLight* and CBT. As predicted, results indicated that *MindLight* is as effective as CBT in the prevention of anxiety. The CI approach showed affirmatively that *MindLight* was non-inferior to CBT over the course of the study for total anxiety symptoms reported by children and parents. *MindLight* showed a larger decrease in child reported personalized anxiety symptoms at post-test and 6-month FU. LGCM analyses demonstrated that children who played *MindLight* showed the same significant decrease in anxiety symptoms compared to those who received CBT. Three- and 6-month follow-up assessments indicated that improvements were sustained based on both child and parent reports of anxiety measures. Moderation analyses showed that improvements were sustained to a somewhat lesser extent for children who were in the *MindLight* condition and had the highest amount of weekly game time. A possible explanation for this might be that these children were less engaged than the other children were, because *MindLight* might have been different than the games they normally play and therefore the effect of *MindLight* might be smaller (Glenn et al. 2013). Taken together, these results show that *MindLight* is an effective anxiety prevention program for at-risk children.

In trials assessing non-inferiority, it is essential that the effect of the gold standard—in this case CBT—is comparable to previous trials. Accordingly, in the current study, the CBT condition yielded effects in line with a previous indicated prevention trial (van Starrenburg et al. 2017). Furthermore, efficacy results for *MindLight* were comparable to those of an initial RCT (Schoneveld et al. 2016). Importantly, both *MindLight* and CBT demonstrated medium within group effect sizes, which corresponds or exceeds effect sizes reported in recent meta-analyses (e.g., Mychailyszyn et al. 2012).

Current results counter a main concern about applied games: that the acquired skills learned through playing a game may not transfer to children’s everyday lives (Girard et al. 2013). First, the measures we used focused on reports of functioning in real-life situations and not on *MindLight* or CBT specifically. For example, statements on self- and parent reports were “I [my son/daughter] am afraid in the dark” and “I [my son/daughter] worry what other people think of me.” Thus, children and parent reports that the anxiety-regulation skill children learned in *MindLight* are not restricted to the game context, but seem to transfer to children’s everyday lives. Second, the fact that not only the children themselves but also their parents reported anxiety decreases and that these improvements were maintained up to 6 months imply transference. This finding moves the applied games field forward as most studies focus only on immediate or short-term improvement. Moreover, the exposure training that is embedded in *MindLight* resembles the more transdiagnostic technique of interoceptive exposure, in which people are exposed to, and made aware of, the physical sensations of anxiety rather than specifying particular anxiety-inducing situations. It seems that children in the *MindLight* group may have learned to regulate their physiological arousal generally and appear to use this skill in their daily lives.

As outlined above, stigma, accessibility, and non-motivating programs prevent children and parents from seeking help or cause them to drop out of conventional prevention programs. In the current study, dropout rates did not differ between the programs. They were equally low in *MindLight* and CBT, because the supervisors (Masters students and psychologists) worked hard to keep attrition in both groups as low as possible. However, in the context of “real world,” implementation where games like *MindLight* could be accessible not only during research protocols but also at home; it may still be that applied games are less likely to show high attrition rates. In addition, when looking into the reasons why children did not want to continue the allocated program, differences between *MindLight* and CBT appeared. Parents of children who dropped out of the CBT program expressed that it took too much time, a reason not mentioned by parents of children who discontinued *MindLight*. This highlights possibly a relative advantage of *MindLight* beyond the first-line treatment of choice for anxiety disorders (CBT): less children might drop out of the program because of time investment issues.

### Children’s Program Ratings

The second aim of the study was to test the emotion-inducing and motivational features of *MindLight*. An important finding was that children rated *MindLight* equally anxiety inducing as CBT. Both programs were rated as anxiety evoking (well above the middle of the scale), which is a prerequisite for children to be able to practice their emotion-regulation skills and for exposure techniques to work. In addition, *MindLight* was rated as equally difficult as CBT. When a game is too difficult, children often experience performance anxiety and give up easily. In contrast, when a game is too easy, children become bored and may lose interest quickly (Nakamura and Csikszentmihalyi 2002). Overall, children rated the difficulty level somewhere in the middle of the scale, suggesting that *MindLight* (and CBT) hit the “sweet spot” of challenge and learning.

Contrary to expectations, children found *MindLight* as appealing as CBT. Both were rated as moderately appealing for themselves and others. It may be that children liked CBT because they got personal attention and it was delivered in a group setting with like-minded peers. In *MindLight*, children were asked to play on their own, at their own pace. This lack of social connection may have made *MindLight* less fun. Given that the majority of gaming is now social (Lenhart et al. 2008), the constrained and individual nature of their game play might have impeded their feelings of autonomy and relatedness and consequently their motivation to play (Ryan and Deci 2000).

Lastly, children rated CBT as more relevant to their daily life than *MindLight*. In CBT, children created their own personal anxiety hierarchy, based on which they chose exercises to practice regulating their anxiety. Children were explicitly told to think about what they do in the CBT sessions, practice the skills through homework assignments in their everyday life, and reflect on those “real-life” practice sessions. *MindLight*, on the other hand, has no such meta-cognitive exercises. The game does not explicitly, and regularly, remind children to practice the skills they learn in the game in their everyday experiences. This was an explicit design decision, aimed to decrease the didactic nature that often significantly diminishes the “fun factor” of most “serious games.” However, as a result, children may have rated *MindLight* as less relevant. It is important to note, however, that *MindLight* was still considered modestly relevant; the children did not rate the game as irrelevant. More critically, our results suggest that this meta-cognizing and explicit didactic exercises that ask children to take what they learn in a training session and apply it to “real life” may not be necessary to produce similar positive improvements as CBT.

### Limitations and Future Directions

Expectations about intervention effects are an important source of potential bias. To equalize expectations across conditions, children and parents were told that both programs were aimed at teaching coping skills in stressful situations. This framing, however, could have primed them to believe that the programs could improve children’s anxiety and hence biased their reports. Future studies could use, in addition to multiple informants, diverse types of measures to assess whether children change in the way they behaviorally cope with, and physiologically regulate, their anxiety.

A clear strength of the current study was the inclusion of a gold-standard active control condition instead of a no-contact or wait-list control group. RCTs are designed to test whether a certain intervention is effective, but they do not inform us about the mechanisms by which the intervention works. An important future step in this line of research is to examine underlying mechanisms by which games like *MindLight* might impact anxiety outcomes. Questions about mechanisms of change could be addressed in dismantling studies (Bell et al. 2013) in which one component of *MindLight* (e.g., neurofeedback, exposure, or attention-bias modification) is removed and the full version is compared to the dismantled version. Despite the call for dismantling studies for over two decades (Kendall et al. 1997) and their feasibility for childhood anxiety interventions (Whiteside et al. 2015), no studies have been conducted in which the full version of an anxiety prevention program is compared with a version missing one or select few components. Games provide a particularly promising avenue for this precise type of research, given their inherent modularity (Granic et al. 2014).

We are strongly encouraged by the findings of the current trial. However, we see this study not as the end of a develop and evaluation process, but the beginning of a promising and challenging approach. As part of that beginning, it is critical to note that most applied games and digital interventions that are developed and tested in a research setting stay in the scientific community, belying the main purpose of their development in the first place: large, scalable impact at low cost (Hollis et al. 2017). One of the reasons for the lack of implementation success might be absence of a systematic strategy for effective dissemination of evidence-based applied games (Gehring et al. 2017). Our Games for Emotional and Mental Health (GEMH) lab is at the early stages of building this strategic framework which includes (a) a replicable methodology by which games for mental health can be co-developed with partners in diverse disciplines including design, engineering and art; (b) an index of resources essential for not only successful development, but also dissemination and/or commercialization and the digital infrastructure required to maintain these interventions; and (c) a set of rationale for applying diverse research approaches (e.g., playtesting, user research, RCTs, experimental designs, qualitative interviews) that test not just for game design elements, outcomes and mechanisms, but also track the success of commercial uptake and other dissemination markers (www.gemhlab.com).

Ultimately, it may not be necessary to compete with the best commercial AAA games on the market to have an impact on young people’s mental health with applied games. Applied games can co-exist with purely education-focused games, just as documentaries co-exist with Hollywood blockbusters, each appealing to individuals for different, and some overlapping, reasons. What does seem to be necessary, however, is for youth to be part of the design and development process so that our games are relevant, appealing, and optimally engaging to their target audience, increasing the probability that they will also be shared with family and friends. Finally, it may be important for scientists to take a more proactive role in engaging commercial industry and making the case for the financial, as well as health, benefits of providing beautiful, entertaining, and scientifically validated mental health tools.

## Conclusion

The current study adds to the growing research on applied games for mental health and shows that these games hold potential as alternative delivery models of therapeutic techniques in mental health prevention. In this non-inferiority RCT, the applied game *MindLight* was shown to be as effective as conventional CBT in reducing child- and parent-reported anxiety levels in 8- to 12-year-old at-risk children. These improvements were maintained at 3- and 6-month follow-ups. Furthermore, *MindLight* and CBT were rated equally anxiety inducing, difficult, and appealing. Given that there are no clinicians or teachers involved and overhead costs associated with the game are non-existent, *MindLight* seems a more cost-effective alternative than traditional anxiety intervention and prevention programs. In terms of school programs, applied games, and *MindLight* specifically, can easily be added to the toolbox of effective prevention approaches already in place in these contexts. Children with concerns about their own capacities to cope with anxiety may be provided with the choice of the delivery model (games or group face-to-face programs), potentially decreasing stigma, increasing their motivation to participate, and ultimately improving mental health outcomes across a broader range of children.

## Electronic supplementary material

ESM 1 (DOCX 18 kb).

ESM 2 (DOCX 14 kb).

ESM 3 (DOCX 14 kb).

ESM 4 (DOCX 17 kb).

## References

[CR1] Asselmann E, Beesdo-Baum K (2015). Predictors of the course of anxiety disorders in adolescents and young adults. Current Psychiatry Reports.

[CR2] Bar-Haim Y, Morag I, Glickman S (2011). Training anxious children to disengage attention from threat: A randomized controlled trial. Journal of Child Psychology and Psychiatry.

[CR3] Beesdo K, Knappe S, Pine DS (2009). Anxiety and anxiety disorders in children and adolescents: Developmental issues and implications for DSM-V. Psychiatric Clinics of North America.

[CR4] Beidas RS, Mychailyszyn MP, Podell JL, Kendall PC (2013). Brief cognitive-behavioral therapy for anxious youth: The inner workings. Cognitive and Behavioral Practice.

[CR5] Bell EC, Marcus DK, Goodlad JK (2013). Are the parts as good as the whole? A meta-analysis of component treatment studies. Journal of Consulting and Clinical Psychology.

[CR6] Brown-Jacobsen AM, Wallace DP, Whiteside SPH (2011). Multimethod, multi-informant agreement, and positive predictive value in the identification of child anxiety disorders using the SCAS and ADIS-C. Assessment.

[CR7] Chen FN, Curran PJ, Bollen KA, Kirby J, Paxton P (2008). An empirical evaluation of the use of fixed cutoff points in RMSEA test statistic in structural equation models. Sociological Methods & Research.

[CR8] de Haan AM, Boon AE, de Jong J, Hoeve M, Vermeiren R (2013). A meta-analytic review on treatment dropout in child and adolescent outpatient mental health care. Clinical Psychology Review.

[CR9] Flannery-Schroeder E, Choudhury MS, Kendall PC (2005). Group and individual cognitive-behavioral treatments for youth with anxiety disorders: 1-year follow-up. Cognitive Therapy and Research.

[CR10] Fleming TM, Bavin L, Stasiak K, Hermansson-Webb E, Merry SN, Cheek C (2017). Serious games and gamification for mental health: Current status and promising directions. Frontiers in Psychiatry.

[CR11] Gehring ND, McGrath P, Wozney L, Soleimani A, Bennett K, Hartling L (2017). Pediatric eMental healthcare technologies: A systematic review of implementation foci in research studies, and government and organizational documents. Implementation Science.

[CR12] Girard C, Ecalle J, Magnan A (2013). Serious games as new educational tools: How effective are they? A meta-analysis of recent studies. Journal of Computer Assisted Learning.

[CR13] Glenn D, Golinelli D, Rose RD, Roy-Byrne P, Stein MB, Sullivan G (2013). Who gets the most out of cognitive-behavioral therapy for anxiety disorders?: The role of treatment dose and patient engagement. Journal of Consulting and Clinical Psychology.

[CR14] Granic I, Lobel A, Engels RCME (2014). The benefits of playing video games. American Psychologist.

[CR15] Green AC, Hunt C, Stain HJ (2012). The delay between symptom onset and seeking professional treatment for anxiety and depressive disorders in a rural Australian sample. Social Psychiatry and Psychiatric Epidemiology.

[CR16] Hollis C, Falconer CJ, Martin JL, Whittington C, Stockton S, Glazebrook C, Davies EB (2017). Annual research review: Digital health interventions for children and young people with mental health problems—a systematic and meta-review. Journal of Child Psychology and Psychiatry.

[CR17] James, A. C., James, G., Cowdrey, F. A., Soler, A., & Choke, A. (2015). Cognitive behavioural therapy for anxiety disorders in children and adolescents. *Cochrane Database of Systematic Reviews.*10.1002/14651858.CD004690.pub4. 10.1002/14651858.CD004690.pub4 PMC6491167 25692403

[CR18] Jones E, Jarvis P, Lewis JA, Ebbutt AF (1996). Trials to assess equivalence: The importance of rigorous methods. British Medical Journal.

[CR19] Kazdin AE (2015). Technology-based interventions and reducing the burdens of mental illness: Perspectives and comments on the special series. Cognitive and Behavioral Practice.

[CR20] Kendall PC, Flannery-Schroeder E, Panichelli-Mindel SM, Southam-Gerow M, Henin A, Warman M (1997). Therapy for youths with anxiety disorders: A second randomized clinical trial. Journal of Consulting and Clinical Psychology.

[CR21] Kendall PC, Hedtke KA (2006). Cognitive-behavioral therapy for anxious children: Therapist manual.

[CR22] Kendall PC, Robin JA, Hedtke KA, Suveg C, Flannery-Schroeder E, Gosch E (2005). Considering CBT with anxious youth? Think exposures. Cognitive and Behavioral Practice.

[CR23] Lenhart, A., Kahne, J., Middaugh, E., Macgill, A. R., Evans, C., & Vitak, J.(2008) Teen, video games, and civics: Teens' gaming experiences are diverse and include significant social interaction and civic engagement. Retrieved from http://www.pewinternet.org/2008/09/16/teens-video-games-and-civics/

[CR24] Mukolo A, Heflinger CA (2011). Factors associated with attributions about child health conditions and social distance preference. Community Mental Health Journal.

[CR25] Muris P, Merckelbach H, Mayer B, Prins E (2000). How serious are common childhood fears?. Behaviour Research and Therapy.

[CR26] Muris P, Schmidt H, Merckelbach H (2000). Correlations among two self-report questionnaires for measuring DSM-defined anxiety disorder symptoms in children: The Screen for Child Anxiety Related Emotional Disorders and the Spence Children’s Anxiety Scale. Personality and Individual Differences.

[CR27] Mychailyszyn MP, Brodman DM, Read KL, Kendall PC (2012). Cognitive-behavioral school-based interventions for anxious and depressed youth: A meta-analysis of outcomes. Clinical Psychology-Science and Practice.

[CR28] Nakamura J, Csikszentmihalyi M, Snyder CR, Lopez SJ (2002). The concept of flow. Handbook of positive psychology.

[CR29] Owens M, Stevenson J, Hadwin JA, Norgate R (2012). Anxiety and depression in academic performance: An exploration of the mediating factors of worry and working memory. School Psychology International.

[CR30] Pardee CS, Colder CR, Bowker JC (2014). Dynamic associations among alcohol use and anxiety symptoms in early adolescence. Psychology of Addictive Behaviors.

[CR31] Piaggio G, Elbourne DR, Pocock SJ, Evans SJW, Altman DG, CONSORT Group (2012). Reporting of noninferiority and equivalence randomized trials extension of the CONSORT 2010 statement. Journal of the American Medical Association.

[CR32] Price J, Budzynski T, Budzynski T, Kogan Budzynski H, Evans JR, Abarbanel A (2009). Anxiety, EEG patterns, and neurofeedback. Introduction to quantitative EEG and neurofeedback: Advanced theory and applications.

[CR33] Ramsawh HJ, Chavira DA (2016). Association of childhood anxiety disorders and quality of life in a primary care sample. Journal of Developmental and Behavioral Pediatrics.

[CR34] Ryan RM, Deci EL (2000). Self-determination theory and the facilitation of intrinsic motivation, social development, and well-being. American Psychologist.

[CR35] Salloum A, Johnco C, Lewin AB, McBride NM, Storch EA (2016). Barriers to access and participation in community mental health treatment for anxious children. Journal of Affective Disorders.

[CR36] Scholten H, Malmberg M, Lobel A, Engels RC, Granic I (2016). A randomized controlled trial to test the effectiveness of an immersive 3D video game for anxiety prevention among adolescents. PloS One.

[CR37] Schoneveld EA, Malmberg M, Lichtwarck-Aschoff A, Verheijen GP, Engels RCME, Granic I (2016). A neurofeedback video game (*MindLight*) to prevent anxiety in children: A randomized controlled trial. Computers in Human Behavior.

[CR38] Spence SH (1997). Structure of anxiety symptoms among children: A confirmatory factor-analytic study. Journal of Abnormal Psychology.

[CR39] Spence SH (1998). A measure of anxiety symptoms among children. Behaviour Research and Therapy.

[CR40] Spence, S. H. (2013). T-Scores. Retrieved from https://www.scaswebsite.com/index.php?p=1_9

[CR41] Stockings EA, Degenhardt L, Dobbins T, Lee YY, Erskine HE, Whiteford HA, Patton G (2016). Preventing depression and anxiety in young people: A review of the joint efficacy of universal, selective and indicated prevention. Psychological Medicine.

[CR42] van Starrenburg ML, Kuijpers RC, Kleinjan M, Hutschemaekers GJ, Engels RC (2017). Effectiveness of a cognitive behavioral therapy-based indicated prevention program for children with elevated anxiety levels: A randomized controlled trial. Prevention Science.

[CR43] Whiteside SPH, Ale CM, Young B, Dammann JE, Tiede MS, Biggs BK (2015). The feasibility of improving CBT for childhood anxiety disorders through a dismantling study. Behaviour Research and Therapy.

[CR44] Whiteside SPH, Brown AM (2008). Exploring the utility of the Spence Children’s Anxiety Scales parent- and child-report forms in a North American sample. Journal of Anxiety Disorders.

[CR45] World Health Organization. (2012). *Making health services adolescent friendly: Developing national quality standards for adolescent friendly health services.* Retrieved from http://apps.who.int/iris/bitstream/10665/75217/1/9789241503594_eng.pdf

