# Supplementary material for: Preventing Childhood Anxiety Disorders: Is an Applied Game as Effective as a Cognitive Behavioral Therapy-Based Program?
Source: Prev Sci. 2017 Sep 27;19(2):220–32. doi: 10.1007/s11121-017-0843-8 (PMC5801383; doi:10.1007/s11121-017-0843-8)
Supplement: Supplementary file 1 — (DOCX 18 kb). [file 11121_2017_843_MOESM1_ESM.docx]

**Supplemental Table A**

Table A

*Similarities on Age, Weekly Game Time, and Expectations at Pretest by Program*

| Measure | Statistic | *MindLight* | CBT | Test result |
| --- | --- | --- | --- | --- |
| Age | Mean (*SD*) | 9.87 (1.16) | 10.07 (1.16) | *t*(162) = 1.08 |
| Weekly game time | Mean (*SD*) | 8.97 (9.24) | 7.95 (7.20) | *t*(161) = -0.79 |
| *MindLight* expectation | Mean (*SD*) | 6.13 (2.11) | 6.10 (2.00) | *t*(163) = -0.12 |
| CBT expectation | Mean (*SD*) | 5.81 (2.28) | 5.90 (1.94) | *t*(161) = 0.28 |
| Sex | *n* girls (%) | 50 (58.1) | 53 (60.2) | χ^2^(1) = 0.08 |

*Note*. All test results were non-significant.
