# Supplementary material for: Preventing Childhood Anxiety Disorders: Is an Applied Game as Effective as a Cognitive Behavioral Therapy-Based Program?
Source: Prev Sci. 2017 Sep 27;19(2):220–32. doi: 10.1007/s11121-017-0843-8 (PMC5801383; doi:10.1007/s11121-017-0843-8)
Supplement: Supplementary file 2 — (DOCX 14 kb). [file 11121_2017_843_MOESM2_ESM.docx]

**Supplemental Table B**

*Pre-test and Change in Anxiety Symptoms Over the Study (Completers Only Sample)*

| Assessment | MindLight | CBT | Mean difference^a^ | SD | 95 % CI | |
| --- | --- | --- | --- | --- | --- | --- |
| Total child | | | | | |  |
| Pre-test | 0.98 | 0.99 |  |  |  | |
| Post-test – pre-test^b^ | -0.24 | -0.25 | 0.01 | 0.35 | [-0.04, 0.06]^c^ | |
| 3-months FU – pre-test^b^ | -0.32 | -0.34 | 0.03 | 0.42 | [-0.06, 0.9]^c^ | |
| 6-months FU – pre-test^b^ | -0.41 | -0.36 | -0.05 | 0.42 | [-0.12, 0.01]^c^ | |
| N | 82 | 83 |  |  |  | |
| Personalized child | | | | | |  |
| Pre-test | 1.39 | 1.31 |  |  |  | |
| Post-test – pre-test ^b^ | -0.32 | -0.19 | -0.13 | 0.47 | [-0.21, -0.06]^d^ | |
| 3-months FU – pre-test ^b^ | -0.41 | -0.37 | -0.04 | 0.54 | [-0.12, 0.05]^c^ | |
| 6-months FU – pre-test ^b^ | -0.53 | -0.37 | -0.16 | 0.56 | [-0.25, -0.07]^d^ | |
| n | 82 | 83 |  |  |  | |
| Total mother | | | | | |  |
| Pre-test | 0.51 | 0.50 |  |  |  | |
| Post-test – pre-test ^b^ | -0.10 | -0.09 | -0.01 | 0.17 | [-0.03, 0.02]^c^ | |
| 3-months FU – pre-test ^b^ | -0.12 | -0.13 | 0.01 | 0.17 | [-0.01, 0.04]^c^ | |
| 6-months FU – pre-test ^b^ | -0.13 | -0.16 | 0.03 | 0.20 | [-0.00, 0.06]^c^ | |
| n | 79 | 78 |  |  |  | |
| Total father | | | | | |  |
| Pre-test | 0.47 | 0.46 |  |  |  | |
| Post-test – pre-test ^b^ | -0.09 | -0.08 | -0.01 | 0.17 | [-0.04, 0.02]^c^ | |
| 3-months FU – pre-test ^b^ | -0.12 | -0.12 | -0.01 | 0.17 | [-0.04, 0.02]^c^ | |
| 6-months FU – pre-test ^b^ | -0.14 | -0.16 | 0.01 | 0.19 | [-0.02, 0.04]^c^ | |
| n | 67 | 66 |  |  |  | |

*Note*. CI = confidence interval.

^a^ A negative difference is a difference in favor of MindLight. ^b^ A negative score means a decrease in the severity of symptoms. ^c^ The 95 % CI of the difference in symptom change lies entirely between the equivalence margins of -0.16 and + 0.16 points, indicating equivalence of MindLight and CBT. ^d^ The 95 % CI of the difference in symptom change lies entirely to the left of zero, indicating significant differences in favor of MindLight.
