# Supplementary material for: Preventing Childhood Anxiety Disorders: Is an Applied Game as Effective as a Cognitive Behavioral Therapy-Based Program?
Source: Prev Sci. 2017 Sep 27;19(2):220–32. doi: 10.1007/s11121-017-0843-8 (PMC5801383; doi:10.1007/s11121-017-0843-8)
Supplement: Supplementary file 3 — (DOCX 14 kb). [file 11121_2017_843_MOESM3_ESM.docx]

**Supplemental Table C**

*Initial Level (Intercept), Change (Linear Slope Component) and Rate of Change (Quadratic Slope Component) in Anxiety Symptoms on Condition (Completers Only Sample)*

|  | Intercept | |  | Linear slope | |  | Quadratic slope | |  |  |  |  |  |
| --- | --- | --- | --- | --- | --- | --- | --- | --- | --- | --- | --- | --- | --- |
|  | *B* | *p* |  | *B* | *p* |  | *B* | *p* |  | χ^2^ (*df*) | *p* | CFI | RMSEA |
| Linear growth model |  |  |  |  |  |  |  |  |  |  |  |  |  |
| Total child |  |  |  |  |  |  |  |  |  | 47.40 (8) | <.001 | 0.69 | 0.17 |
| Personalized child |  |  |  |  |  |  |  |  |  | 30.51 (8) | <.001 | 0.84 | 0.13 |
| Total mother |  |  |  |  |  |  |  |  |  | 40.03 (8) | <.001 | 0.89 | 0.16 |
| Total father |  |  |  |  |  |  |  |  |  | 31.14 (8) | <.001 | 0.94 | 0.15 |
| Quadratic growth model |  |  |  |  |  |  |  |  |  |  |  |  |  |
| Total child | 0.98 | <.001 |  | -1.19 | <.001 |  | 0.95 | <.001 |  | 2.55 (2) | .280 | 1.00 | 0.04 |
| Personalized child | 1.35 | <.001 |  | -1.38 | <.001 |  | 1.07 | <.001 |  | 0.07 (2) | .967 | 1.00 | 0.00 |
| Total mother | 0.50 | <.001 |  | -0.45 | <.001 |  | 0.35 | .001 |  | 3.62 (5) | .606 | 1.00 | 0.00 |
| Total father | 0.46 | <.001 |  | -0.40 | <.001 |  | 0.26 | .005 |  | 1.39 (2) | .498 | 1.00 | 0.00 |
| Program as predictor |  |  |  |  |  |  |  |  |  |  |  |  |  |
| Total child | -0.02 | .780 |  | 0.25 | .415 |  | -0.48 | .157 |  | 3.73 (3) | .292 | 1.00 | 0.04 |
| Personalized child | 0.07 | .304 |  | -0.12 | .774 |  | -0.16 | .768 |  | 6.07 (3) | .108 | 0.98 | 0.08 |
| Total mother | 0.00 | .902 |  | 0.01 | .947 |  | 0.06 | .769 |  | 4.42 (6) | .620 | 1.00 | 0.00 |
| Total father | 0.01 | .842 |  | -0.06 | .672 |  | 0.13 | .476 |  | 1.62 (3) | .656 | 1.00 | 0.00 |

*Note.* Total child = total anxiety child report; Personalized child = personalized anxiety child report; Total mother = total anxiety mother report; Total father = total anxiety father report.
