# Supplementary material for: Preventing Childhood Anxiety Disorders: Is an Applied Game as Effective as a Cognitive Behavioral Therapy-Based Program?
Source: Prev Sci. 2017 Sep 27;19(2):220–32. doi: 10.1007/s11121-017-0843-8 (PMC5801383; doi:10.1007/s11121-017-0843-8)
Supplement: Supplementary file 4 — (DOCX 17 kb). [file 11121_2017_843_MOESM4_ESM.docx]

**Supplemental Table D**

*Initial Level (Intercept), Change (Linear Slope Component) and Rate of Change (Quadratic Slope Component) in Anxiety Symptoms on Condition and Moderators (Completers Only Sample)*

| Predictor | Intercept | |  | Linear slope | |  | Quadratic slope | |  |  |  |  |  |
| --- | --- | --- | --- | --- | --- | --- | --- | --- | --- | --- | --- | --- | --- |
| Anxiety symptoms | *B* | *p* |  | *B* | *p* |  | *B* | *p* |  | χ^2^ (*df*) | *p* | CFI | RMSEA |
| Age |  |  |  |  |  |  |  |  |  |  |  |  |  |
| Total child | 0.04 | .434 |  | -0.15 | .397 |  | 0.15 | .492 |  | 4.15 (5) | .528 | 1.00 | 0.00 |
| Personalized child | 0.01 | .893 |  | -0.02 | .951 |  | 0.13 | .730 |  | 6.59 (5) | .253 | 0.99 | 0.04 |
| Total mother | -0.03 | .477 |  | 0.21 | .052 |  | -0.33 | .041 |  | 7.68 (8) | .466 | 1.00 | 0.00 |
| Total father | -0.03 | .391 |  | 0.07 | .470 |  | -0.05 | .644 |  | 5.38 (5) | .371 | 1.00 | 0.02 |
| Sex |  |  |  |  |  |  |  |  |  |  |  |  |  |
| Total child | 0.11 | .471 |  | 0.00 | 1.00 |  | -0.40 | .595 |  | 8.15 (5) | .148 | 0.99 | 0.06 |
| Personalized child | 0.16 | .379 |  | -0.24 | .808 |  | -0.20 | .862 |  | 17.11 (5) | .004 | 0.95 | 0.12 |
| Total mother | 0.10 | .188 |  | -0.06 | .813 |  | 0.20 | .482 |  | 6.37 (8) | .605 | 1.00 | 0.00 |
| Total father | 0.15 | .044 |  | -0.15 | .655 |  | 0.14 | .746 |  | 1.61 (5) | .900 | 1.00 | 0.00 |
| Expectation |  |  |  |  |  |  |  |  |  |  |  |  |  |
| Total child | -0.01 | .776 |  | -0.20 | .176 |  | 0.27 | .078 |  | 9.28 (5) | .098 | 0.98 | 0.07 |
| Personalized child | 0.05 | .222 |  | -0.42 | .044 |  | 0.52 | .095 |  | 9.75 (5) | .083 | 0.98 | 0.08 |
| Total mother | 0.01 | .461 |  | -0.01 | .881 |  | -0.01 | .889 |  | 8.28 (8) | .407 | 1.00 | 0.02 |
| Total father | -0.03 | .078 |  | -0.07 | .461 |  | 0.15 | .195 |  | 6.12 (8) | .634 | 1.00 | 0.00 |
| Weekly game time |  |  |  |  |  |  |  |  |  |  |  |  |  |
| Total child | 0.01 | .149 |  | -0.04 | .166 |  | 0.06 | .130 |  | 5.06 (5) | .408 | 1.00 | 0.01 |
| Personalized child | 0.02 | .073 |  | -0.06 | .084 |  | 0.10 | .022 |  | 4.27 (5) | .511 | 1.00 | 0.00 |
| Total mother | 0.00 | .799 |  | 0.01 | .706 |  | -0.01 | .416 |  | 7.97 (8) | .437 | 1.00 | 0.00 |
| Total father | 0.00 | .449 |  | 0.02 | .127 |  | -0.04 | .082 |  | 4.22 (8) | .837 | 1.00 | 0.00 |

*Note.* Total child = total anxiety child report; Personalized child = personalized anxiety child report; Total mother = total anxiety mother report; Total father = total anxiety father report.
